# Objection to New Bread

**Published:** 1892-10

**Authors:** 


### OBJECTION TO NEW BREAD.

New bread, if it does not actually cause dyspepia, which is more than likely very frequently the case, is sure to aggravate that trouble. Really the only part of fresh bread fit for the stomach of man is the crust. Dyspeptics at least have learned to appreciate this fact, and many of them eat bread only after it has been toasted. But that process even is well nigh useless when applied to new bread ; and, unless it is done differently than by the most of housekeepers, it is simply a waste of time. Bread fit for toasting must be two or three days old. Instead of submitting the slices to the heat of live coals, a much better way is to bake them in a moderately hot oven. They should be allowed to remain until they are slightly brown all through. Toast made in this way crumbles easily, and is in a state most favorable for digestion; whereas new bread or toast sodden on the inside, when it enters the stomach soon forms into one solid mass of dough, upon the outside only of which can the digestive fluids act. On the other hand, properly made toast forms a spongy mass, in and out of which these fluids can pass freely and do their work speedily. And not only is this toast easily digestible, but it actually favors the digestion of a number of other foods.

